# Alanine Represses γ-Aminobutyric Acid Utilization and Induces Alanine Transaminase Required for Mitochondrial Function in *Saccharomyces cerevisiae*

**DOI:** 10.3389/fmicb.2021.695382

**Published:** 2021-08-04

**Authors:** Dariel Márquez, Ximena Escalera-Fanjul, Mohammed el Hafidi, Beatriz Aguirre-López, Lina Riego-Ruiz, Alicia González

**Affiliations:** ^1^Departamento de Bioquímica y Biología Estructural, Instituto de Fisiología Celular, Universidad Nacional Autónoma de México, Mexico, Mexico; ^2^Theoretical Biophysics, Humboldt-Universität zu Berlin, Berlin, Germany; ^3^Departamento de Biomedicina Cardiovascular, Instituto Nacional de Cardiología Ignacio Chávez, Mexico, Mexico; ^4^División de Biología Molecular, Instituto Potosino de Investigación Científica y Tecnológica (IPICYT), San Luis Potosí, México

**Keywords:** GABA shunt, stress response, respiratory metabolism, mitochondrial genes, transcriptional coregulators

## Abstract

The γ-aminobutyric acid (GABA) shunt constitutes a conserved metabolic route generating nicotinamide adenine dinucleotide phosphate (NADPH) and regulating stress response in most organisms. Here we show that in the presence of GABA, *Saccharomyces cerevisiae* produces glutamate and alanine through the irreversible action of Uga1 transaminase. Alanine induces expression of alanine transaminase (*ALT1*) gene. In an *alt1*Δ mutant grown on GABA, alanine accumulation leads to repression of the *GAD1*, *UGA1*, and *UGA2* genes, involved in the GABA shunt, which could result in growth impairment. Induced *ALT1* expression and negative modulation of the GABA shunt by alanine constitute a novel regulatory circuit controlling both alanine biosynthesis and catabolism. Consistent with this, the GABA shunt and the production of NADPH are repressed in a wild-type strain grown in alanine, as compared to those detected in the wild-type strain grown on GABA. We also show that heat shock induces alanine biosynthesis and *ALT1*, *UGA1*, *UGA2*, and *GAD1* gene expression, whereas an *uga1*Δ mutant shows heat sensitivity and reduced NADPH pools, as compared with those observed in the wild-type strain. Additionally, an *alt1*Δ mutant shows an unexpected alanine-independent phenotype, displaying null expression of mitochondrial *COX2*, *COX3*, and *ATP6* genes and a notable decrease in mitochondrial/nuclear DNA ratio, as compared to a wild-type strain, which results in a petite phenotype. Our results uncover a new negative role of alanine in stress defense, repressing the transcription of the GABA shunt genes, and support a novel Alt1 moonlighting function related to the maintenance of mitochondrial DNA integrity and mitochondrial gene expression.

## Introduction

**FIGURE 1:**
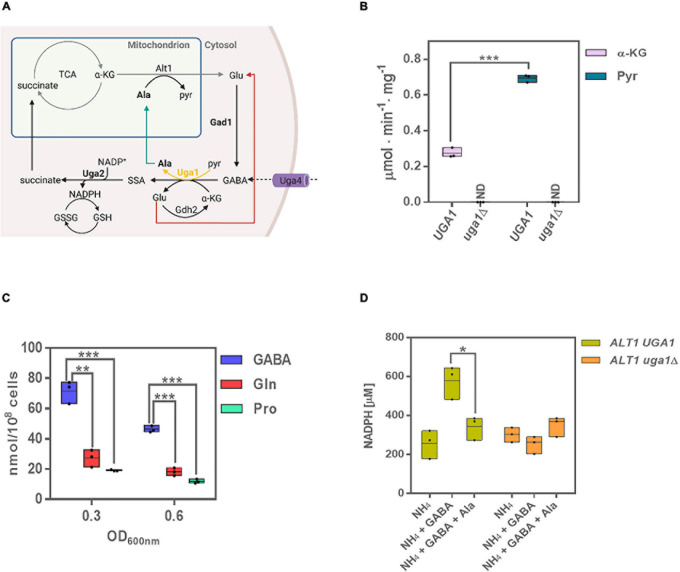
Uga1 transaminase determines GABA-dependent alanine and NADPH biosynthesis. **(A)** The GABA shunt is constituted by three enzymes: Gad1 (glutamate decarboxylase), Uga1 (GABA transaminase), and Uga2 (SSADH). These enzymes are involved in the biosynthesis and catabolism of GABA: α-ketoglutarate (α-kg), originated from the TCA cycle, is converted to glutamate (Glu); this may occur through Alt1 or Uga1; glutamate is then converted to GABA as a result of Gad1-dependent decarboxylation. GABA is also transported to the cell by Uga4 and catabolized by Uga1 to produce succinic semialdehyde (SSA) using α-kg or pyruvate (pyr) as substrates, rendering glutamate or alanine. Lastly, SSA is oxidized to succinate by Uga2 yielding NADPH, and succinate is assimilated by the TCA cycle. The utilization of α-kg or pyr by Uga1 can produce either glutamate or alanine (Ala). Ala is degraded by Alt1; its catabolism generates glutamate, which can be transported to the cytosol and restart the shunt. The glutamate produced by Uga1 may be catabolized by Gdh2 or reassimilated by Gad1. Image created with BioRender.com. **(B)** Enzymatic activity of Uga1 determined in extracts in glucose GABA during exponential phase using α-kg and pyr as substrates. The *uga1*Δ mutant was used as control and grown on ammonium + GABA as this strain does not grow on GABA as the sole nitrogen source. **(C)** Alanine intracellular concentration determined in GABA, glutamine (Gln) or proline (Pro) as nitrogen sources at OD_60__0__*n*__*m*_ = 0.3 and 0.6 during exponential growth phase. **(D)** NADPH determination in glucose as carbon source and the indicated nitrogen sources at OD_60__0__*n*__*m*_ = 0.6 for the wild-type and *uga1*Δ strains. Boxes show individual values (points) and the average (middle line) ± SE of three independent experiments. Asterisks indicate significant differences [***P < 0.001, **P < 0.01, and *P < 0.05, *t*-test (Student)].

The γ-aminobutyric acid (GABA) shunt is a closed-loop process, which produces and consumes GABA (Figure 1A). It converts α-ketoglutarate to succinate, bypassing two reactions of the tricarboxylic acid (TCA) cycle, α-ketoglutarate dehydrogenase and succinate thiokinase (André and Jauniaux, 1990; Bown and Shelp, 1997; Figure 1A). In the absence of an external GABA supply, on ammonium as nitrogen source, glutamate is synthesized through *GDH1* glutamate dehydrogenase as expression of the Gdh3 paralogous enzyme is repressed (Riego et al., 2002; Avendaño et al., 2005). Under these conditions, genes encoding enzymes of the GABA shunt are not induced. Alternatively, when available in the environment, GABA is transported into the cell by three permeases: the specific Uga4 permease (André et al., 1993), the Put4 proline permease (Jauniaux et al., 1987), and the general amino acid permease Gap1 (Grenson et al., 1970), inducing the expression of the enzymes constituting the core of the GABA shunt (André and Jauniaux, 1990; Bown and Shelp, 1997). In natural environments, *Saccharomyces cerevisiae* cells have been isolated from decomposing or damaged fruit, flowering plant nectar, and tree saps (exudates) (Landry et al., 2006; Hittinger, 2013), as well as from insects such as *Drosophila* (Buser et al., 2014), hibernating wasps (Stefanini et al., 2014), and bee hives (Goddard et al., 2010). These microhabitats are rich in sugar but poor in nitrogen, with one or a few nitrogen sources dominating each microhabitat (Ibstedt et al., 2015). However, Phe- and GABA-enriched nectars have been identified in plants pollinated by long-tongued bees and flies (Borghi and Fernie, 2017). Thus, it is feasible that *S. cerevisiae* can be carried from fruit to fruit through insects attracted to GABA-rich nectar plants (Buser et al., 2014).

Intracellular GABA is degraded through the catabolic part of the shunt, which results in succinate production (Ramos et al., 1985; André and Jauniaux, 1990; Coleman et al., 2001; Figure 1A). This process is carried out through the action of the Uga1 transaminase, yielding succinic semialdehyde, which is converted to succinate by the action of the *UGA2*-encoded succinate semialdehyde dehydrogenase (SSADH) producing nicotinamide adenine dinucleotide phosphate (NADPH) (Cao et al., 2013a). Succinate is metabolized via TCA cycle where it is transformed to α-ketoglutarate, which in turn can be converted to glutamate by the Uga1 transaminase of the GABA shunt. Additionally, the fact that Alt1 is mitochondrially located indicates it could contribute to build up the glutamate mitochondrial pool (Figure 1A); however, this is not yet known. In the coming years, research should focus in spatiotemporal biology, shedding light on the role of compartmentalization of metabolic reactions in changing conditions. Assessing the mitochondrial metabolome is challenging, as one should be able to purify intact mitochondria in a short time period. To our knowledge, there is not an established methodology to assess mitochondria metabolism in yeast. Only recently, the mitochondrial proteome was published (Di Bartolomeo et al., 2020). Unfortunately, the protocol established in that study is insufficient to assess mitochondrial metabolism.

Additional biological functions of the GABA shunt such as neurological transmission and antistress mechanisms play an essential role in organism adaptation to changing environments and stress response and have been studied in several organisms (Varju et al., 2001; Petroff, 2002). In *Arabidopsis thaliana* and *S. cerevisiae*, the GABA shunt contributes to stress adaptation (Miyashita and Good, 2008; Cao et al., 2013b); it has been shown that SSADH lack results in the accumulation of reactive oxygen species (ROS) and hypersensitivity to environmental stress (Bouché et al., 2003). *S. cerevisiae* mutants defective in *GAD1*, *UGA1*, or *UGA2* show reduced oxidative stress tolerance and heat sensitivity due to an increased concentration of intracellular ROS (Cao et al., 2013a, b). These observations indicate that the GABA shunt protects from oxidative stress, possibly by counteracting ROS accumulation (Coleman et al., 2001; Cao et al., 2013a). Similar results have been obtained in *A. thaliana* (Cao et al., 2013b).

It has been considered that similar to that observed for the pentose phosphate pathway, which generates NADPH (Kwolek-Mirek et al., 2018), or to lysine biosynthesis inhibition, which leaves untapped NADPH (Olin-Sandoval et al., 2019), yeast GABA shunt NADPH production plays a key role in stress resistance (Grant et al., 1996; Olin-Sandoval et al., 2019). Accordingly, it can be thus considered that Uga2-dependent NADPH production constitutes the basis of the GABA shunt afforded stress resistance (Mead et al., 2013).

The key role of NADPH in oxidative stress prevention depends on glutathione (GSH) reductase, an enzyme catalyzing reduction of GSH disulfide (GSSG), generating the sulfhydryl form of GSH, a key molecule allowing oxidative stress resistance and maintenance of the reducing environment of the cell (Grant et al., 1996; Olin-Sandoval et al., 2019). GSH reductase utilizes NADPH to reduce one molar equivalent of GSSG to two molar equivalents of GSH. Thus, as glutamate priming of the GABA shunt is produced through Uga1-dependent GABA transamination, there is a net gain of NADPH, which in turn contributes to prevent oxidative stress. Accordingly, it has been observed that heat-stressed *Agrostis stolonifera* plants treated with GABA exhibited significantly higher GSH/GSSG ratio than heat-stressed plants without GABA administration (Li et al., 2016). In *S. cerevisiae*, it has been shown that GSH synthesis is induced by heat shock to protect the mitochondrial DNA from oxidative damage that may lead to cell death (Sugiyama et al., 2000).

It has been reported that expression of *UGA1*, *UGA2*, and *UGA4* is exclusively induced by GABA through the combined action of Uga3 and Dal81 transcriptional factors (Vissers et al., 1989). However, neither a repressor signal nor a transcriptional factor negatively regulating the GABA shunt genes or a competitive molecule that could prevent the positive role exerted by Uga3/Dal81 has been identified yet. In *S. cerevisiae*, it has been considered that α-ketoglutarate is the only amino acceptor in the reaction catalyzed by Uga1 (Cao et al., 2013a). Contrastingly, in *A. thaliana*, pyruvate, or α-ketoglutarate acts as an amino acceptor for GABA transamination (Cao et al., 2013b), yet the possibility that alanine or glutamate reaction products could play a regulatory role on the expression of the genes involved in the GABA shunt has not been addressed. However, in *S. cerevisiae*, it has been proposed that alanine accumulation plays a regulatory role negatively affecting expression of the mitochondrial *COX2* gene, encoding cytochrome *c* oxidase, and inducing nuclear *ALT1* expression, which encodes the mitochondrial alanine transaminase (Alt1) (García-Campusano et al., 2009; Peñalosa-Ruiz et al., 2012; Yu et al., 2013).

Alt1 is an alanine transaminase. The reaction catalyzed by transaminases is close to equilibrium, and thus the direction of the reaction depends on substrate or product concentration (Escalera-Fanjul et al., 2017). In the presence of ammonium as the sole nitrogen source, a biosynthetic condition is achieved, and Alt1 contributes 60–75% of the alanine pool (Peñalosa-Ruiz et al., 2012). Conversely, when alanine is the sole nitrogen source, an *alt1Δ* mutant cannot grow, as Alt1 protein constitutes the sole pathway for alanine catabolism (García-Campusano et al., 2009; Peñalosa-Ruiz et al., 2012; Yu et al., 2013).

We found that when *S. cerevisiae* is grown on GABA as the sole nitrogen source, *ALT1* expression was induced, suggesting GABA-dependent alanine biosynthesis, as observed in *A. thaliana* (Cao et al., 2013b). We thus explored whether Uga1 could accept pyruvate as substrate producing succinic semialdehyde (SSA) and alanine. We found that (i) besides α-ketoglutarate, Uga1 alanine transaminase uses pyruvate, generating alanine; (ii) on GABA as the sole nitrogen source, an *alt1*Δ mutant shows impaired growth and accumulates alanine; (iii) in the presence of alanine, expression of the genes whose products form part of the GABA shunt (*UGA1*, *UGA2*, and *GAD1*) is repressed and consequently NADPH production is decreased as compared to that found in the wild-type strain grown on ammonium; and (iv) an *alt1*Δ mutant grown on either ammonium or GABA is respiratory deficient, and null expression of the mitochondrially encoded *COX2*, *COX3*, and *ATP6* genes is observed. However, expression of nuclearly encoded genes involved in oxidative phosphorylation such as *COX6* and *OXA1* displayed wild-type expression profiles. As our results show that alanine is accumulated only on cells grown on GABA, but not on ammonium sulfate, it can be concluded that null expression of mitochondrial genes cannot be attributed to alanine accumulation, suggesting that the mitochondrially located Alt1 (Grandier-Vazeille et al., 2001) could be displaying a moonlighting function, independent of its catabolic role in alanine degradation. In order to further analyze Alt1 role in mitochondrial function, we determined the ratio of mitochondrial DNA (mtDNA) over nuclear DNA (mtDNA/nDNA), finding that in an *alt1*Δ mutant this ratio was twofold to threefold lower as compared to that found in a wild-type strain. These results suggest that Alt1 could be playing a similar role to that of the bifunctional proteins Aco1 and Ilv5 in mtDNA packaging and protection (Chen and Butow, 2005). Thus, it can be proposed that Alt1 moonlighting function could protect cells from stress by safeguarding mtDNA, provoking expression of mitochondrially encoded genes enhancing respiratory metabolism.

## Materials And Methods

### Strains

Supplementary Tables 1, 2, respectively, describe the characteristics of the strains and plasmids used in the present work. Mutant *uga1*Δ (CLA11–738) is a derivative of strain CLA1–2. To construct CLA11–738 strain, the *UGA1* ORF was replaced with the selectable marker *natMX4* cassette by homologous recombination. To obtain *natMX4* cassette, 1,340 bp from plasmid p4339 was amplified using D1 and D2 deoxyoligonucleotides (Supplementary Table 3). This module contained flanking homologous regions in the 5′ UTR from –60 to –1 and in the 3′ UTR from + 1,417 to + 1,476 of *UGA1*. This amplicon was transformed into CLA1-2, and transformants were selected on G418 (200 μg/mL) and confirmed by polymerase chain reaction (PCR) using D3 and D4 primers (Supplementary Table 3).

### Growth Conditions

Strains were grown on minimal medium (MM) containing salts, trace elements, and vitamins according to the formula for yeast nitrogen base (Difco). Glucose (2% wt/vol) or ethanol (2% vol/vol) were used as carbon sources; 7 mM GABA, alanine, proline, and glutamine were used as nitrogen sources or 40 mM ammonium sulfate. Uracil (20 mg/L) was added as auxotrophic requirement when needed. Cells were incubated at 30°C with shaking [250 revolutions/min (rpm)].

### Cell Extract Preparation for GABA Transaminase Enzymatic Assay

Cells were cultured on glucose GABA and were collected at OD_60__0__n__m_ = 0.6. Cells were washed with cold water, suspended in cold extraction buffer (Na_2_HPO_4_ 100 mM, EDTA 10 mM, DTT 0.5 mM; pH 8.0), and mechanically disrupted with glass beads. The resulting extract was centrifuged to eliminate cellular debris (5,000 rpm, 4°C, and 15 min). Protein was measured by the Lowry method (Lowry et al., 1951), using albumin as standard. Enzymatic assay was a modified version of that previously described (Ramos et al., 1985). Buffer A (GABA 10 mM, α-ketoglutarate (α-kg) 16 mM, NADP 0.7 mM, and 3.6 U/mL of glutamate dehydrogenase; Sigma–Aldrich G7882) was used when α-kg was the substrate, and buffer B (GABA 10 mM, pyruvic acid 16 mM, NAD 0.7 mM, and 1 U/mL of alanine dehydrogenase; Sigma–Aldrich A7189) was used when pyruvate was the substrate. Controls were assayed without GABA, and the slope obtained from this negative control was substracted to obtain the real enzymatic specific activity for each assay. Determinations were carried out at 340 nm, 25°C in a Varian Cary 50 spectrophotometer.

### Northern Blot Analysis

Northern blot analysis was carried out as previously described (González et al., 2017). Total yeast RNA was prepared from 200-mL cultures grown to indicated OD_60__0n__m_. Probes to monitor expression of *ALT1*, *UGA1*, *UGA2*, *UGA4*, *GAD1*, *COX2*, *COX3*, *ATP6*, *COX6*, *OXA1*, and *ACT1* were prepared by PCR from CLA1-2 genomic DNA using primers D5-D26 (Supplementary Table 3) and radioactively labeled by α-P^32^ with Random Primer Labeling Kit (Agilent, 300385). Blots were scanned using ImageQuant 5.2 software (Molecular Dynamics).

### Metabolite Extraction and Analysis

Cell extracts were prepared from exponentially growing cultures to indicated OD_60__0__n__m_ in GABA or ammonium sulfate. Samples used for intracellular amino acid determination were treated as previously described (Quezada et al., 2008).

### NADPH Determination

To determine NADPH, cells were grown in indicated conditions. When cultures reached OD_60__0__n__m_ = 0.6 aliquots of 50 mL were taken and centrifuged 5 min at 14,000 rpm. Cells were resuspended in 500 μL of cold buffer N1 (50 mM NaOH and 1 mM EDTA), cells were mechanically disrupted using glass beads. The extract was centrifuged and transferred to a new tube for incubation at 60°C to destroy NAD^+^ and NADP^+^ followed by addition of buffer N2 (100 mM Tris-HCl, pH 8.1; 0.1 M HCl). NADPH was measured enzymatically as follows: phosphate buffer (Na_2_HPO_4_ 100 mM, EDTA 10 mM, DTT 0.5 mM; pH 8.0), 8 mM α-kg, 50 mM NH_4_Cl, and 3.6 U/mL of glutamate dehydrogenase (Sigma–Aldrich G7882). Determinations were carried out at 340 nm, 25°C in a Varian Cary 50 spectrophotometer. The slope of the assay was used to determine total concentration of the NADPH using the molar extinction coefficient (6.22 L/mol⋅cm).

### Nucleosome Scanning Assay

Nucleosome scanning assay (NuSA) was carried out as described previously (Infante et al., 2012); 100-mL cultures in glucose with glutamine, proline, alanine, or GABA were grown to the indicated OD_60__0__n__m_. To determine nucleosome position on *ALT1* promoter, samples were treated as previously described (González et al., 2017). Quantitative PCR (qPCR) analysis was performed using a Corbett Life Science Rotor Gene 6,000 and SYBR Green as dye (2X KAPA SYBR FAST, Invitrogen). Real-time PCR was carried out as follows: 94°C for 5 min (1 cycle), 94°C for 15 s, 58°C for 20 s, and 72°C for 20 s (35 cycles). PCR deoxyoligonucleotides are described in Supplementary Table 4, which amplify from –750 to + 150 bp of *ALT1.* The relative protection was calculated as a ratio to the control of *VCX1* that was amplified using D27 and D28 deoxyoligonucleotides.

### Ethanol Measurement

For ethanol determination cells were grown to the indicated OD_60__0__n__m_ in glucose and GABA or ammonium sulfate as nitrogen sources. Aliquots of 1 mL were withdrawn for supernatant recovery by centrifugation at 14,000 rpm. Ethanol quantification was determined in the supernatant following a previously reported protocol (Calahorra et al., 2012) using the reaction buffer (bicine-KOH 20 mM, pH 9.0; 1 U/mL alcohol dehydrogenase; Sigma–Aldrich A7011). The assay was carried out at 340 nm, 25°C in a Varian Cary 50 spectrophotometer. A standard curve of ethanol to calculate sample concentration was used.

### Thermotolerance Evaluation

Thermotolerance determination was carried out following the protocol described by Cao et al. (2013a). Cultures were grown to OD_60__0__n__m_ = 0.3 in glucose and ammonium sulfate. Cells were harvested by centrifugation at 3,000 rpm, washed with NaCl 0.87%, and resuspended in 1 mL of fresh medium. Aliquots of 100 μL were taken into 1.5-mL tubes for each time point and heated at 37°C or 45°C. After incubation, tubes were placed on ice for 1 min. The cells were then diluted to obtain countable colonies, spread on MM plates, and incubated for 2 days at 30°C. Survival percentage was calculated against the unheated samples.

### Fluorescent Microscopy

Cells were stained with MitoTracker CMXRos (Molecular Probes) according to manufacturer specifications. Confocal images were obtained using a FluoView FV1000 laser confocal system (Olympus) attached/interfaced to an Olympus IX81 inverted light microscope with a 60 × oil-immersion objective (UPLASAPO 60 × O NA:1.35), zoom × 20.0, and 3.5 μm of confocal aperture. The excitation and emission settings were as follows: MitoTracker excitation 543 nm; emission 598 nm, BF 555 nm. The subsequent image processing was carried out with Olympus FluoView FV1000 (version 324 1.7) software.

### Quantification of mtDNA/nDNA Ratio

To quantify the mtDNA, we obtained the amount of mtDNA relative to nDNA. We estimated mtDNA/nDNA ratio by qPCR using different genes of mtDNA: *COX2*, *COX3*, and *ATP6*, and as a nuclear-encoded gene, we selected *COX6*. Total DNA from the indicated strains was extracted twice by phenol/chloroform with 20 μL of NaCl 5 M. Samples were incubated with 20 μg RNase A for 1 h at 37°C. DNA precipitation was carried out with an equal volume of ethanol for 30 min at –20°C and resuspended in nuclease-free water. DNA was quantified by NanoDrop, Thermo Scientific, and samples were diluted at 50 ng/μL for qPCR assay. qPCR was performed using SYBR Green as dye (2X KAPA SYBR FAST, Invitrogen). Conditions for qPCR were as follows: 94°C for 5 min (1 cycle), 94°C for 15 s, 59°C for 30 s, and 72°C for 20 s (30 cycles). We obtained the Ct (cycle threshold) for each sample, and following ΔΔCt method, we calculate the mtDNA/nDNA ratio as described previously (Quiros et al., 2017).

## Results

### *UGA1* Encoded GABA Transaminase: A Pathway for Irreversible Alanine and NADPH Biosynthesis

It has been accepted that in yeast, Uga1 transaminase exclusively uses α-ketoglutarate as α-ketoacid to generate glutamate and succinic semialdehyde (SSA) (Ramos et al., 1985; Cao et al., 2013a). As in plants GABA transaminase is a pyruvate/glyoxylate-dependent enzyme (Miyashita and Good, 2008; Cao et al., 2013b), we determined whether yeast Uga1 GABA transaminase could also use pyruvate (Figure 1A). As Figure 1B shows, Uga1-specific activity was threefold higher with pyruvate than with α-ketoglutarate, indicating that Uga1 can use pyruvate as preferred substrate, and thus, alanine can be generated from GABA. As expected, an *uga1*Δ mutant strain grown on glucose ammonium + GABA showed no enzymatic activity either with α-ketoglutarate or with pyruvate (Figure 1B), indicating GABA transamination is Uga1-dependent. When grown on GABA as the sole nitrogen source, the wild-type strain had a threefold to fourfold higher alanine pool as compared to that found when grown on glutamine or proline as nitrogen sources (Figure 1C). As mentioned above, SSA is readily oxidized through Uga2, preventing its toxic effect (Mekonnen and Ludewig, 2016) and hampering the action of the otherwise freely reversible Uga1 GABA–transaminase reaction (Figure 1A), constraining irreversible alanine and NADPH biosynthesis. As expected, we found that when grown on ammonium–glucose, the wild-type strain generated an NADPH pool, which increased twofold in the presence of GABA, whereas in the presence of both, GABA and alanine, a similar pool to that found on ammonium was observed, suggesting GABA shunt repression on alanine (Figure 1D). An *uga1*Δ mutant strain grown on ammonium or in the presence of GABA or GABA + alanine showed a basal NADPH level similar to that found in the wild-type strain grown on ammonium, indicating that alanine accumulation could result in repression of the GABA shunt function. Taken together, these results indicate that the GABA shunt produces half of the total NADPH pool, observed when the yeast is grown in GABA. The remaining pool, present in either ammonium, GABA, or GABA + alanine in the wild type and *uga1Δ* mutant, must be obtained by the functioning of alternative routes such as the pentose phosphate pathway (Figure 1D).

**FIGURE 2:**
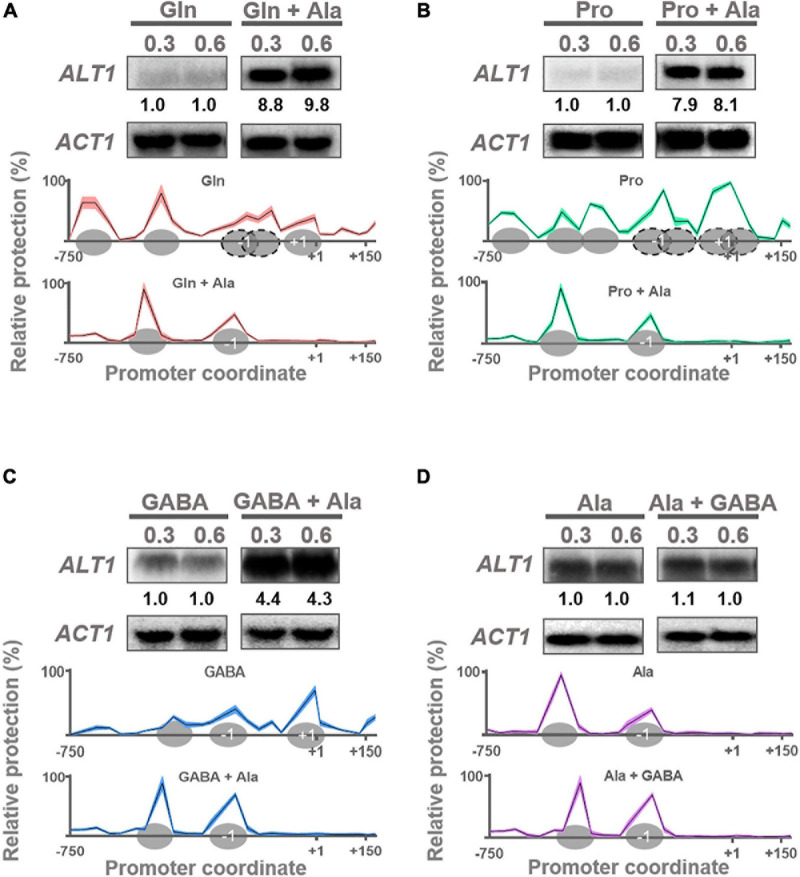
*ALT1* expression is induced in alanine, and nucleosome organization is modified accordingly. Northern blot and NuSA analyses. Expression of *ALT1* and nucleosome promoter occupancy on *ALT1* promoter was determined in **(A)** glutamine (Gln) and glutamine + alanine (Gln + Ala), **(B)** proline (Pro) and proline + alanine (Pro + Ala), **(C)** GABA and GABA + alanine (GABA + Ala), and **(D)** alanine (Ala) and alanine + GABA (Ala + GABA). For Northern blot analyses when each culture reached an OD_60__0__*n*__*m*_ = 0.3, it was divided in two flasks. Alanine 7 mM was added to only one of the cultures. Both cultures (with and without alanine) were incubated at 30°C for additional 15 min. Then, samples of both cultures were taken for RNA extraction. Finally, a second sample of each culture was taken when cells reached an OD_60__0__*n*__*m*_ = 0.6. All samples were prepared for RNA extraction followed by Northern blot assays as described in *Materials and Methods*. Numbers below the blots represent the fold change with respect to samples without alanine **(A–C)** or without GABA **(D)** after quantification and normalization relative to *ACT1*. NuSA samples were obtained as mentioned previously for Northern blot analyses. Only NuSA results obtained at OD_60__0__*n*__*m*_ = 0.6 are presented. Mean values of three independent experiments are shown as solid lines ± SE (shaded area). Ovals over *x*-axis indicate firmly positioned nucleosomes; dotted ovals depict fuzzy nucleosomes. Nucleosomes + 1 and –1 are indicated. ND, not detected.

Glutamate is efficiently metabolized to α-ketoglutarate and ammonium through the NAD-dependent catabolic glutamate dehydrogenase (Gdh2) (Miller and Magasanik, 1990), and alanine is exclusively catabolized by alanine transaminase Alt1 (García-Campusano et al., 2009; Peñalosa-Ruiz et al., 2012), whose expression is alanine-induced (Peñalosa-Ruiz et al., 2012). Here we confirmed that *ALT1* expression is induced only by alanine addition and not by glutamine or proline (Figures 2A,B). However, when GABA was used as the sole nitrogen source, *ALT1*-induced expression was observed (Figure 2C), and alanine addition to the GABA-supplemented media resulted in further induction of *ALT1* expression (Figure 2C). Furthermore, when alanine was provided as the sole nitrogen source, *ALT1*-induced expression was observed (Figure 2D), and GABA addition did not affect *ALT1* expression level, highlighting the fact that alanine constitutes the first described positive modulator of *ALT1* transcription (Figure 2D).

NuSA showed that *ALT1* promoter chromatin organization is closed on both glutamine and proline (Figures 2A,B). In the presence of GABA, where *ALT1* induction occurs, chromatin organization is relaxed (Figure 2C), reaching a fully open structure on GABA plus alanine or when alanine is provided as the sole nitrogen source (Figure 2D), further confirming that *ALT1* expression is alanine induced.

Considering the results presented previously, and the fact than NADPH production is decreased in the presence of GABA + alanine, it can be thus proposed that alanine could constitute a bifunctional regulatory signal determining *ALT1* expression induction and GABA shunt repression. In order to address this proposition, expression of the genes whose products are involved in the operation of the GABA shunt was analyzed.

### Alanine Negatively Regulates the Expression of Genes Encoding Enzymes From the GABA Shunt

**FIGURE 3:**
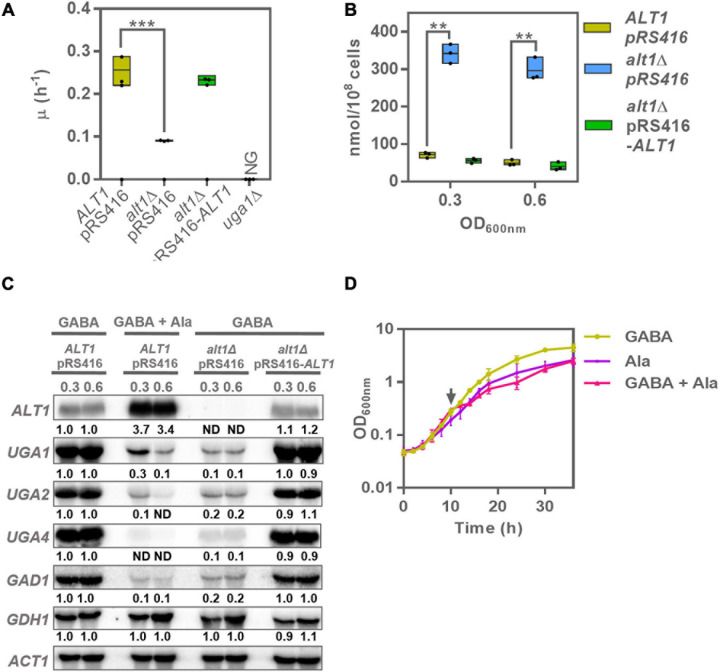
An *alt1*Δ mutant in GABA shows repressed expression of the GABA shunt genes. **(A)** Specific growth rate in glucose GABA of wild-type strain (*ALT1* pRS416), *alt1*Δ mutant strain (*alt1*Δ pRS416), complemented strain (*alt1*Δ pRS416-*ALT1*), and *uga1*Δ strain. **(B)** Intracellular alanine concentration obtained in extracts of cultures grown on glucose + GABA at OD_60__0__*n*__*m*_ = 0.3 and 0.6. Boxes show individual values (points) and the average (middle line) ± SE of three independent experiments. Asterisks indicate significant differences [***P < 0.001 and **P < 0.01, *t*-test (Student)]. **(C)** Northern blot assay prepared from total RNA prepared from glucose GABA grown cells at OD_60__0__*n*__*m*_ = 0.3 and 0.6. Numbers below the blots represent the fold change with respect to the *ALT1* pRS416 strain grown in GABA (rows 1 and 2) after quantification and normalization relative to *ACT1*. **(D)** Growth of the wild-type strain (*ALT1* pRS416) in GABA, alanine, and GABA + alanine. Alanine was added at OD_60__0__*n*__*m*_ = 0.3 (arrow) only for the sample grown in GABA + alanine. Each curve represents the average ± SE of three independent experiments. Abbreviations: NG, no growth; ND, not detected.

In order to analyze whether alanine could negatively regulate the GABA shunt at transcriptional level, we used the *alt1*Δ knockout strain, which as mentioned previously is unable to degrade alanine. We observed that the *alt1*Δ mutation resulted in a threefold growth rate decrease when GABA was used as the sole nitrogen source, as compared to that found in the wild-type strain (Figure 3A). As negative control, we used an *uga1*Δ strain, which is unable to catabolize GABA and does not grow when this amino acid is provided as the sole nitrogen source (Figure 3A; Ramos et al., 1985). As expected, the *alt1*Δ mutant accumulated a threefold to fourfold higher alanine pool when grown on GABA (Figure 3B).

Northern blot analysis showed that in an *alt1*Δ mutant, expression of *UGA4* encoding GABA permease and the shunt core genes *GAD1*, *UGA1*, and *UGA2* was strongly diminished (Figure 3C). A similar result was obtained when total RNA was prepared from wild-type yeast strains grown on GABA + alanine (Figure 3C). As controls, we monitored *ALT1*, whose expression is alanine induced, and *GDH1*, which participates in glutamate biosynthesis, and is not GABA induced, showing wild-type expression levels in the presence of alanine or in an *alt1*Δ mutant. These results indicate that alanine negatively regulates the GABA shunt. Furthermore, when an alanine shock is applied to a culture of the wild-type strain growing on GABA (Figure 3D), growth rate is abruptly diminished confirming that in the presence of alanine, GABA shunt is repressed, and this amino acid cannot be used as nitrogen source. Under this condition (GABA + alanine), growth rate is equivalent to that found when alanine is provided as the sole nitrogen source (Figure 3D). Worth of mention is the fact that although the identification of the mechanism through which alanine participates in *GAD1*, *UGA1*, and *UGA2* expression undoubtedly opens an interesting research question, so far we can only state that alanine-repressing function has not been previously described, and more work beyond the scope of this paper will need to be done to address the mechanism of alanine-dependent repression.

In order to analyze the role of the GABA shunt on stress response, we addressed the question of whether decreasing GABA catabolism impaired the cellular capacity to counteract heat stress.

### *ALT1* and GABA Shunt Expression Are Up-Regulated by Heat Shock Allowing Stress Response

**FIGURE 4:**
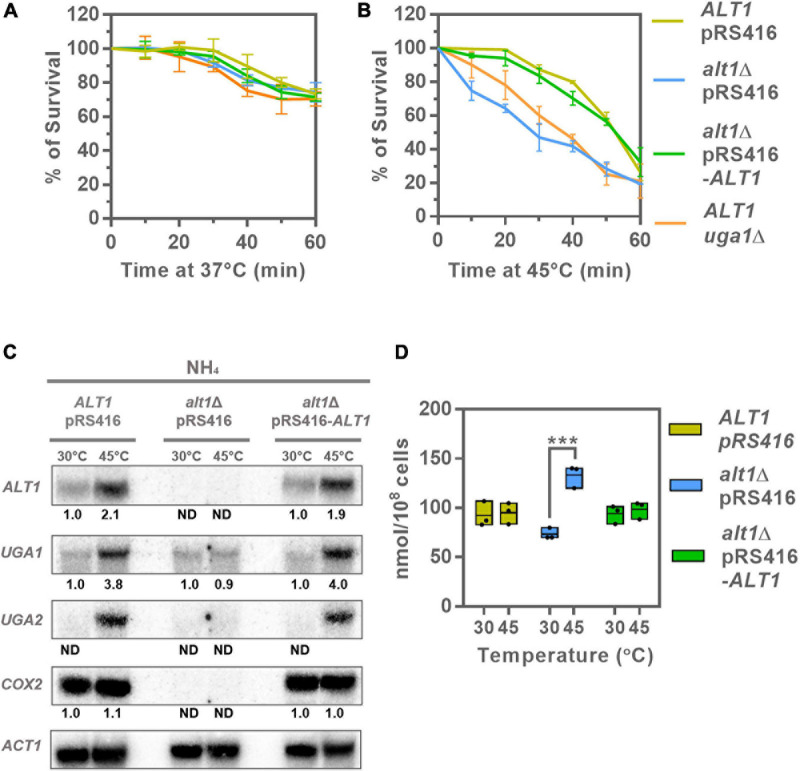
GABA shunt and *ALT1* expression is heat induced: *alt1*Δ and *uga1*Δ display heat sensitivity. Cellular viability, Northern blot, and alanine intracellular pools after heat stress treatment. **(A)** Determination of survival at 37°C from 0- to 60-min calculation of survival percentage was performed as described in section “Materials and Methods.” Data represent average ± SE of three independent experiments. **(B)** Determination of survival at 45°C from 0- to 60-min calculation of survival percentage was performed as described in section “Materials and Methods.” Data represent average ± SE of three independent experiments. **(C)** Northern blot to determine the expression of the GABA shunt genes at 30 and 45°C. Cells were cultured on glucose ammonium (NH_4_) and harvested by centrifugation at OD_60__0__*n*__*m*_ = 0.3 resuspended in glucose ammonium fresh medium, followed by a temperature treatment at 30 or 45°C for 20 min. Total RNA was prepared and used for Northern analysis as described in section “Materials and Methods.” Numbers below the blots represent the fold change with respect to the WT *ALT1* pRS416 strain treated at 30°C (row 1) after quantification and normalization relative to *ACT1*. **(D)** Alanine intracellular pools determined in extracts after a 20-min temperature treatment at 30 and 45°C in the *ALT1* pRS416, *alt1*Δ pRS416, and *alt1*Δ pRS416-*ALT1* strains. Boxes show individual values (points) and the average (middle line) ± SE of three independent experiments. Asterisks indicate significant difference [***P < 0.001, *t*-test (Student)]. Abbreviation: ND, not detected.

As it has been shown that heat shock–dependent ROS production is counteracted by the induction of the GABA shunt (Cao et al., 2013a), we analyzed heat sensitivity of the WT strain, *alt1*Δ pRS416-*ALT1* and *alt1*Δ pRS416 to heat shock at two different temperatures. As Figure 4A shows, after a 37°C heat shock, the wild-type strain, the *alt1*Δ pRS416-*ALT1*, the *alt1*Δ, and *uga1*Δ mutants showed equivalent viability, indicating that after a 37°C heat shock the GABA shunt function is not evoked. However, as Figure 4B shows, a 45°C heat shock showed that the *uga1*Δ mutant displayed a thermosensitive phenotype, indicating that under this condition, functioning of the GABA shunt is compelling for heat stress response. The fact that the *alt1*Δ mutant displayed similar heat sensitivity to that of the *uga1*Δ mutant indicates that alanine accumulation due to Alt1 lack could be repressing expression of the GABA shunt genes, thus hindering heat stress–dependent induction and stress response. To further analyze this matter, the wild-type strain was grown on ammonium–glucose MM at 30°C until the cultures reached OD_60__0__*n*__*m*_ = 0.3, after which they were exposed for 20 min at 45°C. As shown in Figure 4B, after the 20-min treatment, wild-type strain and *alt1*Δ pRS416-*ALT1* mutant displayed 100% viability. Total RNA was extracted, and Northern analysis was carried out. Northern results observed in Figure 4C have been normalized by measuring *ACT1* expression, which is constitutively expressed, affording an indirect measure of the cell number. We can say that for all cases actin concentration was equivalent at 30 or 45°C (Figure 4C).

As Figure 4C shows, *ALT1*, *UGA1*, and *UGA2* increased their expression, indicating these genes were up-regulated under stress conditions. The fact that the wild-type strain and the *alt1*Δ pRS416-*ALT1*–complemented strain show identical phenotypes, reverting that of the *alt1*Δ pRS416 mutant, indicates that reported alanine pools and *ALT1*, *UGA1*, and *UGA2* expression are *ALT1*-dependent.

As expected, *COX2* expression was not regulated by heat shock (Figure 4C), as the mitochondrially encoded subunit II of cytochrome *c* oxidase (Complex IV) is not involved in stress response.

*ALT1* heat-induced expression could not be due to an indirect effect of alanine-dependent expression induction because, as Figure 4D shows, alanine concentration in the wild-type strain is similar at 30 and 45°C. When the *alt1*Δ pRS416 mutant was heat-shocked, *UGA1* and *UGA2* expression induction was not observed (Figure 4C). Accordingly, after heat shock, an *alt1Δ* mutant displayed an alanine pool, which was twofold higher than that found in the heat-stressed wild-type or in the *alt1*Δ pRS416-*ALT1* strains. This result indicates that heat shock response was strongly counteracted by alanine-mediated repression, highlighting the dominant role of alanine-mediated repression over the heat-induced GABA shunt stress response. Worth of mention is the fact that an *alt1*Δ mutant at 30°C accumulates a lower alanine pool as compared to that found in the wild-type strain under the same condition (Figure 4D) because, as mentioned previously, under this biosynthetic condition alanine is mainly synthesized by the *ALT1*-encoded alanine transaminase (Peñalosa-Ruiz et al., 2012; Escalera-Fanjul et al., 2017).

As it had been proposed that *COX2* expression was negatively influenced by alanine intracellular concentration (Yu et al., 2013), we assayed *COX2* expression as an additional control (Figure 4C). Heat shock treatment showed that *COX2* expression was not induced even at 45°C (Figure 4C). Most interesting was the observation that an *alt1*Δ mutant completely abolished *COX2* expression at either 30 or 45°C, conditions that, respectively, show low or high alanine intracellular pools (Figures 4C,D). These observations indicate that *COX2* repression was not alanine-dependent. It can be thus concluded that *COX2* repression, although being Alt1-dependent, is alanine-independent. To further analyze the physiological effect of Alt1 lack, we analyzed the phenotype of an *alt1*Δ mutant grown under respiratory conditions.

### *alt1*Δ Mutants Are Impaired in Respiratory Metabolism: mtDNA/nDNA Ratio Is Decreased, and *COX2*, *COX3*, and *ATP6* Expression Is Null

**FIGURE 5:**
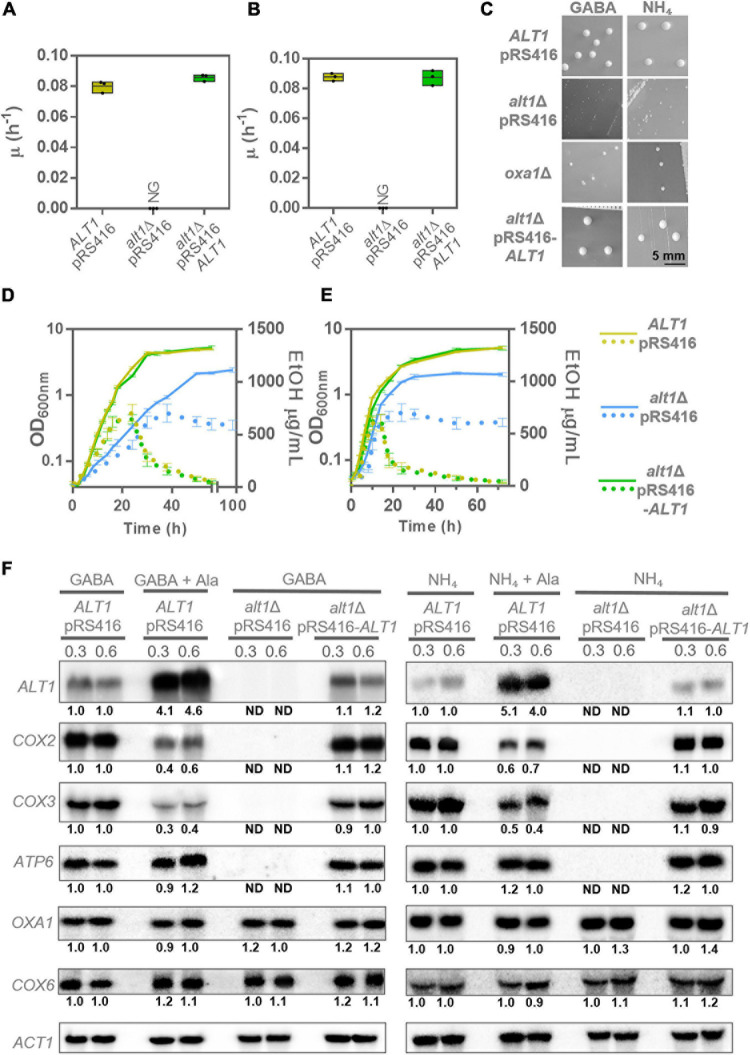
*alt1*Δ mutant shows petite phenotype and severely decreased expression of various mitochondrial genes. **(A)** Specific growth rate in ethanol GABA. **(B)** Specific growth rate in ethanol ammonium. Boxes show individual values (points) and the average (middle line) ± SE of three independent experiments. **(C)** Colony size in glucose + GABA (GABA) and glucose + ammonium (NH_4_). To obtain colony size, plates were incubated at 30°C during 4 days in the case of glucose GABA (GABA) and 3 days in the case of glucose ammonium (NH_4_). **(D)** Cells were cultured to monitor biomass (solid lines) and ethanol concentration (dotted lines) in glucose + GABA. Each line represents the average ± SE of three independent experiments. **(E)** Cells were cultured to monitor biomass (solid lines) and ethanol concentration (dotted lines) in glucose + ammonium. Each line represents the average ± SE of three independent experiments. **(F)** Northern blot in glucose + GABA (GABA) and glucose + ammonium (NH_4_) to determine the expression of mitochondrial genes (*COX2*, *COX3*, and *ATP6*) in the presence of alanine (+Ala) and in the *alt1*Δ mutant at OD_60__0__*n*__*m*_ = 0.3 and 0.6. Alanine was added to observe the effect on the mitochondrial gene expression. *COX6* and *OXA1* are nuclear genes and were tested to determine any possible effect of alanine addition or *ALT1* absence. Numbers below the blots represent the fold change with respect to the *ALT1* pRS416 strain grown in GABA or ammonium (rows 1 and 2 of each Northern assay) after quantification and normalization relative to *ACT1*. Abbreviations: NG, no growth; ND, not detected.

As Figures 5A,B show, an *alt1*Δ mutant did not grow on ethanol, exhibiting a petite phenotype when grown on either glucose + GABA or glucose + ammonium sulfate (Figure 5C). Additionally, it was found that an *alt1*Δ mutant produces ethanol, which after glucose exhaustion was unable to consume on either GABA or ammonium (Figures 5D,E). The fact that alanine concentration showed contrasting levels, high on GABA and low on ammonium (Figures 1C, 4B), strongly suggests that the lack of *ALT1*, and not alanine accumulation, exerts the negative modulation of respiratory metabolism. Contrastingly, previous results obtained by another group led them to conclude that decreased expression of *COX2* in an *alt1*Δ mutant was due to alanine accumulation (Yu et al., 2013).

To further analyze Alt1 role on the regulation of mitochondrially encoded genes, in addition to *COX2*, we determined the expression of the mitochondrially encoded gene *COX3*, as both proteins form part of respiratory Complex IV (Geier et al., 1995), as well as the mitochondrial gene *ATP6*, which encodes the subunit a of the F0 sector of FIF0 ATP synthase (Devenish et al., 2000). Worth of mention is the fact that mitochondrial genes are expressed as polycistronic transcripts. Accordingly, *COX2*, *COX3*, and *ATP6* belong to different transcriptional units whose expression is controlled by independent promoters. Thus, we analyzed three of the four polycistronic units encoding proteins from the mitochondrial genome. As shown in Figure 5F, *COX2*, *COX3*, and *ATP6* expression was abolished in an *alt1*Δ mutant grown on either GABA or ammonium, as compared to that observed in the presence of alanine, suggesting that an Alt1 moonlighting property (Singh and Bhalla, 2020), and not an alanine-dependent regulatory effect as that observed for the GABA shunt genes, was responsible for impaired *COX2*, *COX3*, and *ATP6* expression. We further analyzed the expression of the nuclear-encoded *COX6* and *OXA1* genes. Cox6, together with Cox2 and Cox3, forms part of cytochrome *c* oxidase Complex IV (Geier et al., 1995). Oxa1 constitutes a mitochondrial inner membrane insertase, mediating the anchorage of both mitochondrial- and nuclear-encoded proteins from the mitochondrial matrix into the inner membrane (Szyrach et al., 2003). As can be seen in Figure 5F, *OXA1* and *COX6* expression showed wild-type levels.

Worth of mention is the fact that, as shown in Figure 5F, and although as mentioned previously, *COX2* and *COX3* expression was not fully repressed by alanine, a slight decrease on RNA levels was observed in the presence of alanine (GABA + alanine or ammonium sulfate + alanine). Conversely, *ATP6* expression was not affected under these conditions (Figure 5F). This indicates that, in addition to the strong *alt1Δ* negative effect on mtDNA concentration, alanine could play a discrete role as *COX2* and *COX3* negative coregulator. Most important was the finding that *OXA1* and *COX6* nuclear-encoded genes showed wild-type expression in either the presence of alanine or in an *alt1Δ* mutant, indicating that only the expression of some mitochondrial respiratory chain genes is affected by alanine concentration.

**FIGURE 6:**
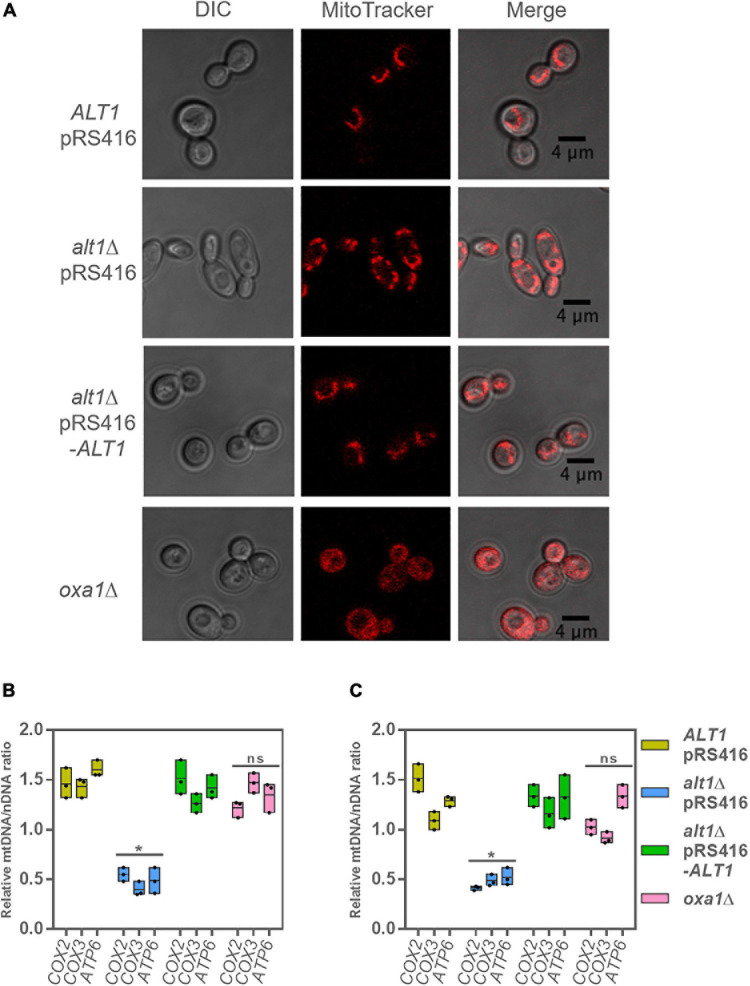
*alt1*Δ mutant shows diminished mtDNA vs. nDNA concentration. **(A)** Mitochondrial staining with MitoTracker CMXRos to determine integrity of mitochondria for indicated strains. Cells were harvested in glucose + GABA and collected at OD_60__0__*n*__*m*_ = 0.6. Estimation of mtDNA/nDNA ratio by qPCR using *COX2*, *COX3*, and *ATP6* as mitochondrial genes; this determination is relative to *COX6* encoded nuclear gene in **(B)** glucose + GABA and **(C)** glucose + ammonium. Boxes show individual values (points) and the average (middle line) ± SE of three independent experiments. Asterisk indicate significant differences *P < 0.05, *t-*test (Student). Abbreviation: ns, not significant.

Considering that the components of both respiratory Complexes II and III are nuclearly encoded, we decided to evaluate whether membrane potential could be developed in an *alt1Δ* mutant determining if MitoTracker CMXRos stained mitochondria. As shown in Figure 6C, the wild-type strain, the *alt1*Δ pRS416-*ALT1*, and the *alt1Δ* mutant cells show stained mitochondria, indicating that nuclearly encoded proteins constituting Complexes II and III were functional and capable to generate membrane potential. These results confirm that lack of Alt1 affects only mtDNA integrity and not that of nDNA. As expected, in the *oxa1Δ* mutant, which as mentioned earlier lacks the mitochondrial inner membrane insertase, the MitoTracker CMXRos was retained in the cytoplasm as this mutant strain is unable to generate membrane potential, due to impaired function of all respiratory complexes (Figure 6A).

In order to further analyze the role of Alt1 in mitochondrial function, we determined the ratio of mtDNA/nDNA using *COX6* as nuclear control. As shown in Figures 6B,C, when DNA was quantified in samples obtained from GABA or ammonium grown cells, the mtDNA/nDNA ratio showed a twofold to threefold decrease when qPCR *COX2*, *COX3*, or *ATP6* amplification was carried out in samples obtained from the *alt1Δ* mutant as compared to that found in the wild-type strain, indicating that Alt1 could affect mtDNA stability. This could result in impaired transcription, as observed for *COX2*, *COX3*, and *ATP6* (Figure 5F).

## Discussion

### Alanine Is a Versatile Signaling Molecule Inducing *ALT1* Expression and Repressing That of the GABA Shunt Genes

**FIGURE 7:**
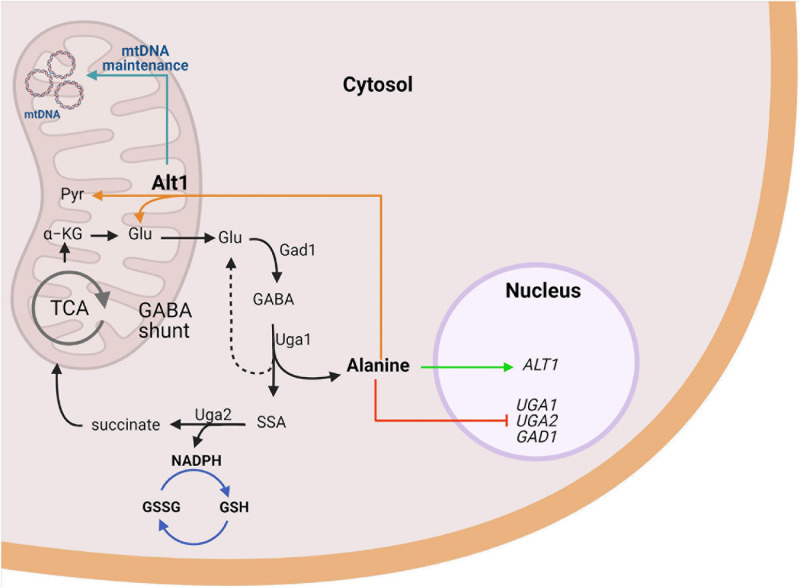
Alanine represses GABA utilization and induces alanine transaminase required for mitochondrial function in *S. cerevisiae*. Uga1, the sole catabolic pathway degrading GABA in yeast, yields glutamate (dashed line) and alanine (solid line). Alanine exerts a dual coregulator role on the expression, being positive for *ALT1* and negative for *UGA* genes. Glutamate produced by Uga1 or alanine catabolism through Alt1 maintains the GABA shunt. GABA metabolism results in net NADPH production, which promotes defense against oxidative stress through GSH restoring. Furthermore, our results suggest Alt1 displays a moonlight function independent of its alanine transaminase capacity, a role that allows the adequate mitochondrial functioning through mtDNA maintenance due to an unidentified mechanism. Image created with BioRender.com.

Yeasts are able to adjust their metabolic capacity in response to variations in the nutrient supply of the culture medium. The recognition of changes in environmental conditions and the ability to adapt to these changes are essential for the viability of yeast cells (Conrad et al., 2014). Our results show that in *S. cerevisiae*, GABA degradation through Uga1 irreversibly leads to alanine biosynthesis (Figure 7). Therefore, we analyzed whether alanine could constitute a regulatory cue controlling the function of the GABA shunt. We demonstrated that alanine acts as a corepressor of *GAD1*, *UGA1*, *UGA2*, and *UGA*4 expression, resulting in the negative regulation of the GABA shunt. Thus GABA, which is the substrate of Uga1 transaminase, positively regulates expression of the genes involved in the shunt (Talibi et al., 1995; Sylvain et al., 2011), whereas alanine, the product of its degradation, represses expression of the shunt. Additionally, our results show that alanine induces *ALT1* expression, whose encoded transaminase plays a key role in the catabolism of the alanine pool generated through Uga1 GABA transaminase (Figure 7). When grown on GABA, an *alt1Δ* mutant accumulates fourfold higher alanine levels, as compared to a wild-type strain. Under this condition, genes whose products are involved in the GABA shunt are repressed, and growth on GABA is severely hindered. Thus, alanine constitutes a regulatory cue determining both its biosynthesis and catabolism (Figure 7). In agreement with previous results (Ramos et al., 1985; Cao et al., 2013a, b), we found that when ammonium was used as the sole nitrogen source, in a wild-type strain GABA shunt gene expression was induced after heat shock. However, in an *alt1Δ* mutant exposed to heat shock treatment, induction of GABA shunt gene expression was minor compared to the one observed in a wild-type strain. These results indicate that, under this condition, accumulated alanine represses the shunt, counteracting GABA shunt heat-mediated induction, diminishing yeast heat stress tolerance.

For the first time, we have identified a critical role for alanine as a negative regulator of the GABA shunt gene expression (*GAD1*, *UGA1*, *UGA2*) and potentially a general regulator of the cellular responses to stress (Figure 7). Presented results highlight the fact that alanine constitutes a versatile positive (*ALT1*) or negative (*GAD1*, *UGA1*, *UGA2*, and *UGA4*) coregulator indicating its complex role in transcriptional regulation (Figure 7). That alanine can play a transcriptional regulatory role had been previously suggested (Usaite et al., 2006). It has been found that yeast cells grown on alanine-limited cultures show peculiar whole-genome transcript profiles as compared to those found on glutamine- or ammonium-limited conditions, highlighting the fact that the nitrogen source has an important influence on the generation of a selective gene transcriptome in yeasts. Furthermore, it was shown that alanine could evoke a peculiar transcriptional profile, as alanine grown cells showed increased transcript levels of the *IRC7* encoding cysteine desulfhydrase involved in cysteine catabolic process to pyruvate, *ALT1* encoding alanine transaminase, and *GDH2* encoding the glutamate degrading dehydrogenase (Figure 1A; Usaite et al., 2006). These results indicate a role for alanine as modulator of carbon and nitrogen metabolism. Further analysis of the role of alanine in transcriptional modulation will have to be performed in order to identify the transcriptional regulator(s) that influences alanine role as corepressor of the GABA shunt genes and *ALT1* expression induction.

### Alt1, a Moonlighting Protein

The mitochondrial genome of *S. cerevisiae* contains 35 genes, including those that code for polypeptides, small (rns) and large (rnl) rRNA subunits, and a variable number of tRNA genes (Turk et al., 2014). The core set of polypeptide encoding genes comprise ATP synthase subunits (Atp6, Atp8, and Atp9), cytochrome *c* oxidase subunits (Cox1, Cox2, and Cox3), and apocytochrome b (Cob1) (Turk et al., 2014), all of which are involved in oxidative phosphorylation. These seven genes are transcribed from four different promoters; *COX2*, *COX3*, *COX1*, *ATP8*, and *ATP6* are distributed in three of them, whereas *ATP9* and *COB1* are transcribed from a fourth and a fifth promoter (Turk et al., 2014). Therefore, presented results show expression from three of the five polycistronic promoters. We found that *alt1*Δ mutants show a petite phenotype, being unable to grow under respiratory conditions and unable to use the ethanol produced during the fermentative phase on glucose based media. As the aforementioned petite phenotype is observed in the *alt1Δ* mutant grown on either GABA, where a high alanine pool is accumulated, or on ammonium sulfate, where the intracellular pool of this amino acid constitutes only 20% of that found in the wild-type strain (Peñalosa-Ruiz et al., 2012; Escalera-Fanjul et al., 2017), it can be concluded that this phenotype is independent of alanine accumulation, but Alt1-dependent. In GABA or ammonium grown cultures, expression of *COX3*, *COX2*, and *ATP6* is completely abolished, indicating that Alt1 plays an additional role in mitochondrial gene regulation, not related to alanine accumulation.

The proposed Alt1 function, unrelated to its role as transaminase, could constitute a novel, moonlighting function. Mitochondrial DNA (mtDNA) is packaged with specific proteins in compact DNA–protein complexes named mitochondrial nucleoids (mt-nucleoids). Abf2 is a mtDNA-binding protein, which plays a prominent role in the packaging of mtDNA into the nucleoid structure (Basu et al., 2020) that protects mtDNA against damage (Miyakawa, 2017). The analysis of an *abf2Δ* mutant showed that when grown on media containing a fermentable carbon source, cells lose mtDNA and become respiratory deficient (petite) (Miyakawa, 2017). Most interesting has been the finding that *ILV5* constitutes a multicopy suppressor of the *abf2Δ* petite phenotype (Bateman et al., 2002). *ILV5* encodes an acetohydroxy acid reductoisomerase, which participates in branched-chain amino acid biosynthesis. The involvement of Ilv5 protein in mtDNA stabilization does not depend on the functioning of the branched-chain amino acid biosynthetic pathway, indicating bifunctionality of the Ilv5 protein (Bateman et al., 2002). Although it has been shown that Ilv5 binds DNA *in vitro* (Macierzanka et al., 2008), its exact role in mtDNA compaction remains unknown; however, *ilv5Δ* mutants that lose DNA-binding activity produce petite mutants (Bateman et al., 2002). Whether Alt1 moonlighting role could be related to the group of proteins that pack and protect mt-nucleoids remains to be addressed. In this regard, mitochondrial gene transcription has not been addressed in mutants in which mt-DNA packing is altered; it could be speculated that alteration of mt-DNA organization could result in transcriptional alterations as those observed in the *alt1Δ* mutant. Alt1 moonlighting function could afford additional physiological roles. It could constitute a respiratory control compensating the lack of oxidative stress protection, due to alanine accumulation, diminishing respiratory metabolism and providing conditions for fermentation. These presumed functions remain to be addressed (Figure 7).

## Conculsion

It can be concluded that alanine plays a dual role as negative coregulator of the GABA shunt, and positive coregulator of *ALT1* expression. This function, together with the previously described positive role of GABA as inductor of GABA shunt genes, could constitute an interesting regulatory network ultimately determining the stress response, as well as the intracellular alanine pool concentration not only in yeasts but also in other biological systems. Additionally, it was found that an *alt1Δ* deletion prevents respiratory metabolism by abolishing expression of several mitochondrial genes involved in oxidative phosphorylation. The fact that the expression of the nuclear-encoded genes of oxidative metabolism is not repressed in *alt1Δ* mutants and that Alt1 is localized in the mitochondrial matrix (Grandier-Vazeille et al., 2001) allowed us to propose a moonlighting property for Alt1. Considering that the petite *alt1Δ* mutant showed threefold decreased mtDNA/nDNA as compared to the wild-type strain, it can be proposed that Alt1 could play a role in the organization of mtDNA, simultaneously affecting mtDNA/nDNA concentration, transcription of the mitochondrial genome, and potentially the degradation of its cognate mRNAs, processes that would constitute a most interesting research goal requiring further investigation (Figure 7).

## Data Availability Statement

The raw data supporting the conclusions of this article will be made available by the authors, without undue reservation.

## Author Contributions

DM, XE-F, LR-R, and AG designed research, analyzed data, and wrote the manuscript. DM, XE-F, MH, and BA-L performed research. All authors contributed to the article and approved the submitted version.

## Conflict of Interest

The authors declare that the research was conducted in the absence of any commercial or financial relationships that could be construed as a potential conflict of interest.

## Publisher’s Note

All claims expressed in this article are solely those of the authors and do not necessarily represent those of their affiliated organizations, or those of the publisher, the editors and the reviewers. Any product that may be evaluated in this article, or claim that may be made by its manufacturer, is not guaranteed or endorsed by the publisher.

## References

[B1] André B. Jauniaux J. C. (1990). Nucleotide sequence of the yeast *UGA1* gene encoding GABA transaminase. *Nucleic Acids Res.* 18:3049. 10.1093/nar/18.10.3049 2190186 PMC330839

[B2] André B. Hein C. Grenson M. Jauniaux J. C. (1993). Cloning and expression of the *UGA4* gene coding for the inducible GABA-specific transport protein of *Saccharomyces cerevisiae*. *Mol. Gen. Genet.* 237 17–25. 10.1007/BF00282779 8455553

[B3] Avendaño A. Riego L. DeLuna A. Aranda C. Romero G. Ishida C. (2005). Swi/SNF-GCN5-dependent chromatin remodelling determines induced expression of *GDH3*, one of the paralogous genes responsible for ammonium assimilation and glutamate biosynthesis in *Saccharomyces cerevisiae*. *Mol. Microbiol.* 57 291–305. 10.1111/j.1365-2958.2005.04689.x 15948967

[B4] Basu U. Bostwick A. M. Das K. Dittenhafer-Reed K. E. Patel S. S. (2020). Structure, mechanism, and regulation of mitochondrial DNA transcription initiation. *J. Biol. Chem.* 295 18406–18425. 10.1074/jbc.REV120.011202 33127643 PMC7939475

[B5] Bateman J. M. Perlman P. S. Butow R. A. (2002). Mutational bisection of the mitochondrial DNA stability and amino acid biosynthetic functions of Ilv5p of budding yeast. *Genetics* 161 1043–1052. 10.1093/genetics/161.3.1043 12136009 PMC1462179

[B6] Borghi M. Fernie A. R. (2017). Floral Metabolism of Sugars and Amino Acids: Implications for Pollinators’ Preferences and Seed and Fruit Set. *Plant Physiol.* 175 1510–1524. 10.1104/pp.17.01164 28986424 PMC5717749

[B7] Bouché N. Fait A. Bouchez D. Møller S. G. Fromm H. (2003). Mitochondrial succinic-semialdehyde dehydrogenase of the gamma-aminobutyrate shunt is required to restrict levels of reactive oxygen intermediates in plants. *Proc. Natl. Acad. Sci. U S A.* 100 6843–6848. 12740438 10.1073/pnas.1037532100 PMC164534

[B8] Bown A. W. Shelp B. J. (1997). The Metabolism and Functions of [gamma]-Aminobutyric Acid. *Plant Physiol.* 115 1–5. 10.1104/pp.115.1.1 12223787 PMC158453

[B9] Buser C. C. Newcomb R. D. Gaskett A. C. Goddard M. R. (2014). Niche construction initiates the evolution of mutualistic interactions. *Ecol. Lett.* 17 1257–1264. 10.1111/ele.12331 25041133

[B10] Calahorra M. Sánchez N. S. Peña A. (2012). Characterization of glycolytic metabolism and ion transport of *Candida albicans*. *Yeast* 29 357–370. 22899221 10.1002/yea.2915

[B11] Cao J. Barbosa J. M. Singh N. K. Locy R. D. (2013a). GABA shunt mediates thermotolerance in *Saccharomyces cerevisiae* by reducing reactive oxygen production. *Yeast* 30 129–144. 10.1002/yea.2948 23447388

[B12] Cao J. Barbosa J. M. Singh N. Locy R. D. (2013b). GABA transaminases from *Saccharomyces cerevisiae* and *Arabidopsis thaliana* complement function in cytosol and mitochondria. *Yeast* 30 279–289. 10.1002/yea.2962 23740823

[B13] Chen X. J. Butow R. A. (2005). The organization and inheritance of the mitochondrial genome. *Nat. Rev. Genet.* 6 815–825. 10.1038/nrg1708 16304597

[B14] Coleman S. T. Fang T. K. Rovinsky S. A. Turano F. J. Moye-Rowley W. S. (2001). Expression of a glutamate decarboxylase homologue is required for normal oxidative stress tolerance in *Saccharomyces cerevisiae*. *J. Biol. Chem.* 276 244–250. 10.1074/jbc.M007103200 11031268

[B15] Conrad M. Schothorst J. Kankipati H. N. Van Zeebroeck G. Rubio-Texeira M. Thevelein J. M. (2014). Nutrient sensing and signaling in the yeast *Saccharomyces cerevisiae*. *FEMS Microbiol. Rev.* 38 254–299. 10.1111/1574-6976.12065 24483210 PMC4238866

[B16] Devenish R. J. Prescott M. Roucou X. Nagley P. (2000). Insights into ATP synthase assembly and function through the molecular genetic manipulation of subunits of the yeast mitochondrial enzyme complex. *Biochim. Biophys. Acta* 1458 428–442. 10.1016/s0005-2728(00)00092-x 10838056

[B17] Di Bartolomeo F. Malina C. Campbell K. Mormino M. Fuchs J. Vorontsov E. (2020). Absolute yeast mitochondrial proteome quantification reveals trade-off between biosynthesis and energy generation during diauxic shift. *Proc. Natl. Acad. Sci. U S A.* 117 7524–7535. 10.1073/pnas.1918216117 32184324 PMC7132131

[B18] Escalera-Fanjul X. Campero-Basaldua C. Colón M. González J. Márquez D. González A. (2017). Evolutionary Diversification of Alanine Transaminases in Yeast: Catabolic Specialization and Biosynthetic Redundancy. *Front. Microbiol.* 8:1150. 10.3389/fmicb.2017.01150 28694796 PMC5483587

[B19] García-Campusano F. Anaya V. H. Robledo-Arratia L. Quezada H. Hernández H. Riego L. (2009). ALT1-encoded alanine aminotransferase plays a central role in the metabolism of alanine in *Saccharomyces cerevisiae*. *Can. J. Microbiol.* 55 368–374. 10.1139/w08-150 19396236

[B20] Geier B. M. Schägger H. Ortwein C. Link T. A. Hagen W. R. Brandt U. (1995). Kinetic properties and ligand binding of the eleven-subunit cytochrome-c oxidase from *Saccharomyces cerevisiae* isolated with a novel large-scale purification method. *Eur. J. Biochem.* 227 296–302. 10.1111/j.1432-1033.1995.tb20388.x 7851399

[B21] Goddard M. R. Anfang N. Tang R. (2010). A distinct population of *Saccharomyces cerevisiae* in New Zealand: Evidence for local dispersal by insects and human-aided global dispersal in oak barrels. *Env. Micro* 12 63–73. 10.1111/j.1462-2920.2009.02035.x 19691498

[B22] González J. López G. Argueta S. Escalera-Fanjul X. El Hafidi M. Campero-Basaldua C. (2017). Diversification of Transcriptional Regulation Determines Subfunctionalization of Paralogous Branched Chain Aminotransferases in the Yeast. *Genetics* 207 975–991. 10.1534/genetics.117.300290 28912343 PMC5676234

[B23] Grandier-Vazeille X. Bathany K. Chaignepain S. Camougrand N. Manon S. Schmitter J. M. (2001). Yeast mitochondrial dehydrogenases are associated in a supramolecular complex. *Biochemistry* 40 9758–9769. 10.1021/bi010277r 11502169

[B24] Grant C. M. Collinson L. P. Roe J. H. Dawes I. W. (1996). Yeast glutathione reductase is required for protection against oxidative stress and is a target gene for yAP-1 transcriptional regulation. *Mol. Microbiol.* 21 171–179. 10.1046/j.1365-2958.1996.6351340.x 8843443

[B25] Grenson M. Hou C. Crabeel M. (1970). Multiplicity of the amino acid permeases in *Saccharomyces cerevisiae*. IV. Evidence for a general amino acid permease. *J. Bacteriol.* 103 770–777. 10.1128/JB.103.3.770-777.1970 5474888 PMC248157

[B26] Hittinger C. T. (2013). *Saccharomyces* diversity and evolution: a budding model genus. *Trends Genet.* 29 309–317. 10.1016/j.tig.2013.01.002 23395329

[B27] Ibstedt S. Stenberg S. Bagés S. Gjuvsland A. B. Salinas F. Kourtchenko O. (2015). Concerted Evolution of Life Stage Performances Signals Recent Selection on Yeast Nitrogen Use. *Mol. Biol. Evol.* 32 153–161. 10.1093/molbev/msu285 25349282

[B28] Infante J. J. Law G. L. Young E. T. (2012). Analysis of nucleosome positioning using a nucleosome-scanning assay. *Methods Mol. Biol.* 833 63–87. 10.1007/978-1-61779-477-3_5 22183588

[B29] Jauniaux J. C. Vandenbol M. Vissers S. Broman K. Grenson M. (1987). Nitrogen catabolite regulation of proline permease in *Saccharomyces cerevisiae*. Cloning of the *PUT4* gene and study of *PUT4* RNA levels in wild-type and mutant strains. *Eur. J. Biochem.* 164 601–606. 3552672 10.1111/j.1432-1033.1987.tb11169.x

[B30] Kwolek-Mirek M. Maslanka R. Molon M. (2018). Disorders in NADPH generation via pentose phosphate pathway influence the reproductive potential of the *Saccharomyces cerevisiae* yeast due to changes in redox status. *J. Cell. Biochem.* 2018:28140. 10.1002/jcb.28140 30474881

[B31] Landry C. R. Townsend J. P. Hartl D. L. Cavalieri D. (2006). Ecological and evolutionary genomics of *Saccharomyces cerevisiae*. *Mol. Ecol.* 15 575–591. 10.1111/j.1365-294X.2006.02778.x 16499686

[B32] Li Z. Yu J. Peng Y. Huang B. (2016). Metabolic pathways regulated by γ-aminobutyric acid (GABA) contributing to heat tolerance in creeping bentgrass (*Agrostis stolonifera*). *Sci. Rep.* 6:30338. 10.1038/srep30338 27455877 PMC4960583

[B33] Lowry O. H. Rosebrough N. J. Farr A. L. Randall R. J. (1951). Protein measurement with the Folin phenol reagent. *J. Biol. Chem.* 193 265–275. 10.1016/s0021-9258(19)52451-6 14907713

[B34] Macierzanka M. Plotka M. Pryputniewicz-Drobinska D. Lewandowska A. Lightowlers R. Marszalek J. (2008). Maintenance and stabilization of mtDNA can be facilitated by the DNA-binding activity of Ilv5p. *Biochim. Biophys. Acta* 1783 107–117. 10.1016/j.bbamcr.2007.09.009 18023287

[B35] Mead O. Thynne E. Winterberg B. Solomon P. S. (2013). Characterising the role of GABA and its metabolism in the wheat pathogen *Stagonospora nodorum*. *PLoS One* 8:e78368. 10.1371/journal.pone.0078368 24265684 PMC3827059

[B36] Mekonnen D. W. Ludewig F. (2016). Phenotypic and chemotypic studies using *Arabidopsis* and yeast reveal that GHB converts to SSA and induce toxicity. *Plant Mol. Biol.* 91 429–440. 10.1007/s11103-016-0475-6 27037708

[B37] Miller S. M. Magasanik B. (1990). Role of NAD-linked glutamate dehydrogenase in nitrogen metabolism in *Saccharomyces cerevisiae*. *J. Bacteriol.* 172 4927–4935. 10.1128/jb.172.9.4927-4935.1990 1975578 PMC213147

[B38] Miyakawa I. (2017). Organization and dynamics of yeast mitochondrial nucleoids. *Proc. Jpn. Acad. Ser. B Phys. Biol. Sci.* 93 339–359. 10.2183/pjab.93.021 28496055 PMC5489437

[B39] Miyashita Y. Good A. G. (2008). Contribution of the GABA shunt to hypoxia-induced alanine accumulation in roots of *Arabidopsis thaliana*. *Plant Cell Physiol.* 49 92–102. 10.1093/pcp/pcm171 18077464

[B40] Olin-Sandoval V. Yu J. S. L. Miller-Fleming L. Alam M. T. Kamrad S. Correia-Melo C. (2019). Lysine harvesting is an antioxidant strategy and triggers underground polyamine metabolism. *Nature* 572 249–253. 10.1038/s41586-019-1442-6 31367038 PMC6774798

[B41] Peñalosa-Ruiz G. Aranda C. Ongay-Larios L. Colon M. Quezada H. Gonzalez A. (2012). Paralogous *ALT1* and *ALT2* retention and diversification have generated catalytically active and inactive aminotransferases in *Saccharomyces cerevisiae*. *PLoS One* 7:e45702. 10.1371/journal.pone.0045702 23049841 PMC3458083

[B42] Petroff O. A. (2002). GABA and glutamate in the human brain. *Neuroscientist* 8 562–573. 10.1177/1073858402238515 12467378

[B43] Quezada H. Aranda C. DeLuna A. Hernández H. Calcagno M. L. Marín-Hernández Á, et al. (2008). Specialization of the paralogue *LYS21* determines lysine biosynthesis under respiratory metabolism in *Saccharomyces cerevisiae*. *Microbiology* 154 1656–1667. 10.1099/mic.0.2008/017103-0 18524920

[B44] Quiros P. M. Goyal A. Jha P. Auwerx J. (2017). Analysis of mtDNA/nDNA Ratio in Mice. *Curr. Protoc. Mouse Biol.* 7 47–54. 10.1002/cpmo.21 28252199 PMC5335900

[B45] Ramos F. el Guezzar M. Grenson M. Wiame J. M. (1985). Mutations affecting the enzymes involved in the utilization of 4-aminobutyric acid as nitrogen source by the yeast *Saccharomyces cerevisiae*. *Eur. J. Biochem.* 149 401–404. 10.1111/j.1432-1033.1985.tb08939.x 3888627

[B46] Riego L. Avendaño A. DeLuna A. Rodríguez E. González A. (2002). *GDH1* expression is regulated by *GLN3*, *GCN4*, and *HAP4* under respiratory growth. *Biochem. Biophys. Res. Commun.* 293 79–85. 10.1016/S0006-291X(02)00174-2 12054566

[B47] Singh N. Bhalla N. (2020). Moonlighting Proteins. *Annu. Rev. Genet.* 54 12.1–12.21. 10.1146/annurev-genet-030620-102906 32870732

[B48] Stefanini I. Dapporto L. Legras J. L. Calabretta A. Di Paola M. De Filippo C. (2014). Role of social wasps in *Saccharomyces cerevisiae* ecology and evolution. *Proc. Natl. Acad. Sci. U S A.* 109 13398–13403. 10.1073/pnas.1208362109 PMC3421210 22847440

[B49] Sugiyama K. Kawamura A. Izawa S. Inoue Y. (2000). Role of glutathione in heat-shock-induced cell death of *Saccharomyces cerevisiae*. *Biochem. J.* 352 71–78. 10.1042/bj3520071 11062059 PMC1221433

[B50] Sylvain M. A. Liang X. B. Hellauer K. Turcotte B. (2011). Yeast zinc cluster proteins Dal81 and Uga3 cooperate by targeting common coactivators for transcriptional activation of γ-aminobutyrate responsive genes. *Genetics* 188 523–534. 10.1534/genetics.110.126003 21515579 PMC3176551

[B51] Szyrach G. Ott M. Bonnefoy N. Neupert W. Herrmann J. M. (2003). Ribosome binding to the Oxa1 complex facilitates co-translational protein insertion in mitochondria. *EMBO J.* 22 6448–6457. 10.1093/emboj/cdg623 14657018 PMC291818

[B52] Talibi D. Grenson M. André B. (1995). Cis- and trans-acting elements determining induction of the genes of the gamma-aminobutyrate (GABA) utilization pathway in *Saccharomyces cerevisiae*. *Nucleic Acids Res.* 23 550–557. 10.1093/nar/23.4.550 7899074 PMC306719

[B53] Turk E. M. Das V. Seibert R. D. Andrulis E. D. (2014). The mitochondrial landscape of *Saccharomyces cerevisiae*. *PLoS One* 8:e78105. 10.1371/journal.pone.0078105 24143261 PMC3797045

[B54] Usaite R. Patil K. R. Grotkjaer T. Nielsen J. Regenberg B. (2006). Global transcriptional and physiological responses of *Saccharomyces cerevisiae* to ammonium, L-alanine, or L-glutamine limitation. *Appl. Environ. Microbiol.* 72 6194–6203. 10.1128/AEM.00548-06 16957246 PMC1563674

[B55] Varju P. Katarova Z. Madarász E. Szabó G. (2001). GABA signalling during development: new data and old questions. *Cell Tissue Res.* 305 239–246. 10.1007/s004410100356 11545261

[B56] Vissers S. Andre B. Muyldermans F. Grenson M. (1989). Positive and negative regulatory elements control the expression of the *UGA4* gene coding for the inducible 4-aminobutyric-acid-specific permease in *Saccharomyces cerevisiae*. *Eur. J. Biochem.* 181 357–361. 2653828 10.1111/j.1432-1033.1989.tb14732.x

[B57] Yu S. L. An Y. J. Yang H. J. Kang M. S. Kim H. Y. Wen H. (2013). Alanine-metabolizing enzyme Alt1 is critical in determining yeast life span, as revealed by combined metabolomic and genetic studies. *J. Proteome Res.* 12 1619–1627. 10.1021/pr300979r 23527786

