# Supplementary material for: Alanine Represses γ-Aminobutyric Acid Utilization and Induces Alanine Transaminase Required for Mitochondrial Function in Saccharomyces cerevisiae
Source: Front Microbiol. 2021 Aug 4;12:695382. doi: 10.3389/fmicb.2021.695382 (PMC8371705; doi:10.3389/fmicb.2021.695382)
Supplement: Supplementary file 1 [file Data_Sheet_1.docx]

**Supplementary Table 1.** Strains used in the present work.

| Strain | Genotype | Reference |
| --- | --- | --- |
| *CLA1-2* | *MATα ALT1 ura3 leu2::LEU2* | 1 |
| *CLA1-2-1* | *MATα alt1Δ::kanMX4 ura3 leu2::LEU2* | 2 |
| *CLA11-738* | *MATα ALT1 uga1Δ::natMX4 ura3 leu2::LEU2* | This study |
| *oxa1*Δ | BY4741  *MATa his3Δ1 leu2Δ0 met15Δ0 ura3Δ0 oxa1Δ::HygB* | Provided by Soledad Funes and Maria Clara Avendaño |

| **Plasmids** | **Source** |
| --- | --- |
| p4339 | 3 |
| pRS416 | 4 |
| pRS416::*ALT1* | 5 |

**Supplementary Table 2.** Plasmids used in the present work.

**Supplementary Table 3.** Deoxyoligonucleotides used in the present work.

| Primer | Sequence (5’-3’) | Application |
| --- | --- | --- |
| D1 | TGTGTTACATTACAGAAAGAACAGACAAGAAACCGTCAATAAGAAATATAACTAAGAACA**AGATCTGTTTAGCTTGCCTCGTCCCCGCCG** | Fw from -60 to -1 of *UGA1* and for p4339-natMX4 (sequence in bold) used to generate *uga1*Δ mutant |
| D2 | CAAGATACACATATATAAAGACCAAAAAAGGGAACGTGACACGGCCTCGCTAATATACAA**GAATTCGAGCTCGTTTTCGACACTGGATGG** | Rv from +2417 to +2476 of *UGA1* and for p4339-natMX4 (sequence in bold) used to generate *uga1*Δ mutant |
| D3 | CAATAGTTTTAGGATTTTTCCTTCTCCC | Fw from -103 to -76 of *UGA1* used for confirmation of *uga1*Δ |
| D4 | TCTTTACTGCAGTCACCAAACCCA | Rv from +2561 to 2485 of *UGA1* used for confirmation of *uga1*Δ |
| D5 | AGACCCGTCCTACAGAGACATAGC | Fw from +172 to +195 of *ALT1* used for Northern probe |
| D6 | GCGAGCTTCTTGAACTGCCTTGAA | Rv from +1558 to +1581 of *ALT1* used for Northern probe |
| D7 | GTGAACAATACTACCCAAGAAGAGCC | Fw from +11 to +35 of *UGA1* used for Northern probe |
| D8 | CACATCCACCAACGTTGCAACC | Rv from +1297 to +1318 of *UGA1* used for Northern probe |
| D9 | GTAAGTATTCTAAACCAACTCTAAACGACCC | Fw from +11 to +41 of *UGA2* used for Northern probe |
| D10 | AATCCTGACTCTTTAACACCACCAAATGG | Rv from +1381 to +1406 of *UGA2* used for Northern probe |
| D11 | CCACTGATATCGGTCAGGCTTACC | Fw from +53 to +76 of *UGA4* used for Northern probe |
| D12 | CGATAACATCAGCACCAACGGCTTC | Rv from +1663 to +1687 of *UGA4* used for Northern probe |
| D13 | GATACTTCGTCTCAGGGCATGGC | Fw from +148 to +170 of *GAD1* used for Northern probe |
| D14 | CTTTTCGATTTCATCCGGCGTTGGG | Rv from +1682 to +1707 of *GAD1* used for Northern probe |
| D15 | TGAATGATGTACCAACACCTTATGCATG | Fw from +41 to +68 of *COX2* used for Northern probe |
| D16 | CGATCTTAATTGGCATATTTGCATGACC | Rv from +682 to +709 of *COX2* used for Northern probe |
| D17 | GCCTTCACCATGACCTATTGTAGTATC | Fw from +54 to +80 of *COX3* used for Northern probe |
| D18 | GACTCCTCATCAGTAGAAGACTACGTA | Rv from +781 to +807 of *COX3* used for Northern probe |
| D19 | GCCAGACCTGGTGCTTATCATGC | Fw from +51 to +77 of *COX6* used for Northern probe |
| D20 | GGGAACGCCCAATTCTTGTCTGAC | Rv from +391 to +414 of *COX6* used for Northern probe |
| D29 | GTTTTGCCGGTGACGAC | Fw from +368 to +384 of *ACT1* used for Northern probe |
| D30 | CTTTCGGCAATACCTGGG | Rv from +1227 to +1244 of *ACT1* used for Northern probe |
| D31 | TGCGTGTGCATCCCTACTGA | Fw from +1504 to +1524 of *VCX1* used for NuSA analysis |
| D32 | AAGTGGTCTTCCTTGCCATGA | Rv from +1552 to +1572 of *VCX1*  used for NuSA analysis |

**Supplementary Table 4.** Primers used for nucleosome scanning assays in *ALT1* promoter.

| Name | Sequence 5’ to 3’ | Middle point of PCR (Promoter coordinate) | 5’/3’ end | Amplicon size (bp) |
| --- | --- | --- | --- | --- |
| A1 | Fw ATGATGTTTCAGGGCAAGCTTT  Rv AGAGGAGACCGGTTATACGA | -748 | -796 | 96 |
|  |  |  | -700 |  |
| A2 | Fw AATCTCTTGCACGGTCGCAT  Rv AAAGTAAGTACGCAGGTGTAGTT | -709 | -760 | 102 |
|  |  |  | -658 |  |
| A3 | Fw TCGTATAACCGGTCTCCTCT  Rv ATCTTAAGAGATATGCCGCTTG | -670 | -720 | 101 |
|  |  |  | -619 |  |
| A4 | Fw AACTACACCTGCGTACTTACTTT  Rv TTTGCCTCTCTGCCTTTCTTT | -630 | -681 | 103 |
|  |  |  | -578 |  |
| A5 | Fw CAAGCGGCATATCTCTTAAGAT  Rv ACGAGCCATCCTCTGCAGA | -596 | -641 | 90 |
|  |  |  | -551 |  |
| A6 | Fw TTTGCCTCTCTGCCTTTCTTT  Rv TGCTGTGCAATCCTTGTTTCT | -550 | -599 | 98 |
|  |  |  | -501 |  |
| A7 | Fw TCTGCAGAGGATGGCTCGT  Rv TATGTGTGTGGGGCAGCG | -523 | -568 | 90 |
|  |  |  | -478 |  |
| A8 | Fw AGAAACAAGGATTGCACAGC  Rv TATATGGATTATATAGCCACGAAT | -476 | -520 | 89 |
|  |  |  | -431 |  |
| A9 | Fw CGCTGCCCCACACACATA  Rv ACGTCTAATGGGAAGTGCTA | -436 | -496 | 121 |
|  |  |  | -375 |  |
| A10 | Fw ATTCGTGGCTATATAATCCATATA  Rv AGTGAAAAAAAAACCAGTACTGTAA | -398 | -454 | 113 |
|  |  |  | -341 |  |
| A11 | Fw TAGCACTTCCCATTAGACGT  Rv CTTGGACCCCTTCAGAATGA | -348 | -394 | 92 |
|  |  |  | -302 |  |
| A12 | Fw TTACAGTACTGGTTTTTTTTTCACT  Rv TTGTCTCAACGAAAGGTCGAA | -323 | -374 | 103 |
|  |  |  | -271 |  |
| A13 | Fw TCATTCTGAAGGGGTCCAAG  Rv AACCACAGCAGGTCAGAAGA | -281 | -321 | 80 |
|  |  |  | -241 |  |
| A14 | Fw TTCGACCTTTCGTTGAGACAA  Rv ATCGTTTACAAAGGGCGCAG | -248 | -291 | 87 |
|  |  |  | -204 |  |
| A15 | Fw TCTTCTGACCTGCTGTGGTT  Rv GTAGTAACGGAAGAGCTTTTC | -210 | -260 | 99 |
|  |  |  | -161 |  |
| A16 | Fw CTGCGCCCTTTGTAAACGAT  Rv ACAACTAGAGTAAAATAGCGAGAA | -176 | -223 | 94 |
|  |  |  | -129 |  |
| A17 | Fw GAAAAGCTCTTCCGTTACTAC  Rv AAGCTGGTCCTGTTCTCGA | -136 | -181 | 90 |
|  |  |  | -91 |  |
| A18 | Fw TTCTCGCTATTTTACTCTAGTTGT  Rv TGCGTTCAATTGAGAAGCAGA | -108 | -153 | 91 |
|  |  |  | -62 |  |
| A19 | Fw TCGAGAACAGGACCAGCTT  Rv CAAAATAAAGACTGGGGAAAT | -59 | -109 | 100 |
|  |  |  | -9 |  |
| A20 | Fw TCTGCTTCTCAATTGAACGCA  Rv TGAAGTGATTTTTGGCAGACAGT | +5 | -62 | 133 |
|  |  |  | +71 |  |
| A21 | Fw ATTTCCCCAGTCTTTATTTTGCT  Rv GAGTTATAGAATTACTCACTGTG | +10 | -32 | 84 |
|  |  |  | +52 |  |
| A22 | Fw ACTGTCTGCCAAAAATCACTTCA  Rv TTTCTGCGCTTGAAGTGAGA | +54 | +8 | 92 |
|  |  |  | +100 |  |
| A23 | Fw CACAGTGAGTAATTCTATAACTC  Rv AAAGGAGTAGTGATATGTGGCA | +77 | +29 | 96 |
|  |  |  | +125 |  |
| A24 | Fw TCTCACTTCAAGCGCAGAAA  Rv AATCCAGGAAGAGCTATGTCT | +130 | +80 | 99 |
|  |  |  | +179 |  |
| A25 | Fw TGCCACATATCACTACTCCTTT  Rv AATCCAGGAAGAGCTATGTCT | +154 | +104 | 100 |
|  |  |  | +204 |  |

**References**

1. Quezada, H., et al. (2008). Specialization of the paralogue *LYS21* determines lysine biosynthesis under respiratory metabolism in *Saccharomyces cerevisiae*. Microbiology 154, 1656-1667. doi: 10.1099/mic.0.2008/017103-0
2. García-Campusano, F., et al. (2009). *ALT1*-encoded alanine aminotransferase plays a central role in the metabolism of alanine in *Saccharomyces cerevisiae*. Can. J. Microbiol. 55, 368-374. doi: 10.1139/w08-150.
3. Goldstein, A.L., and McCusker, J.H. (1999). Three new dominant drug resistance cassettes for gene disruption in *Saccharomyces cerevisiae*. Yeast 15, 1541-1553. doi: 10.1002/(SICI)1097-0061(199910)15:14<1541::AID-YEA476>3.0.CO;2-K
4. Sikorski, R.S.,and Hieter, P. (1989). A system of shuttle vectors and yeast host strains designed for efficient manipulation of DNA in *Saccharomyces cerevisiae*. Genetics 122, 19-27.
5. Escalera-Fanjul, X., et al. (2017). Evolutionary Diversification of Alanine Transaminases in Yeast: Catabolic Specialization and Biosynthetic Redundancy. Front. Microbiol. 8, 1150. doi: 10.3389/fmicb.2017.01150.
